# Supplementary material for: Utilization of organic-residues as potting media: Physico-chemical characteristics and their influence on vegetable production
Source: PLoS One. 2024 Jun 11;19(6):e0302135. doi: 10.1371/journal.pone.0302135 (PMC11166280; doi:10.1371/journal.pone.0302135)
Supplement: Attachment — Submitted filename: Response to Reviewers.docx [file pone.0302135.s001.docx]

**Response to Reviewers’ Comments**

**Reference:** PONE-D-24-02845

**Title:** Utilization of organic-residues as potting media: Physico-chemical characteristics and their influence on vegetable production

NOTE: Revised/added text as per review comments has been highlighted in the revised manuscript through track changes.

Authors acknowledge the comments by respectable reviewers and editor to improve the quality of the manuscript. Thank you very much for these efforts and for sparing valuable time.

**Reviewer #1:**

**Query 1. I suggest the author remove abbreviated words from the abstract and revise it. Kindly use full names here.**

**Response:** The abbreviation from the abstract has been removed and revised with full names as suggested.

**Query 2.** **Line 37-40: Kindly revise it. I found some inconsistencies in these sentences.**

**Response:** Sentences have been revised.

**Query 3. I suggest the author revise the keywords and it should be not the same as the title of the manuscript.**

**Response:** Needful has been done.

**Query 4. Line 81: Do not start the sentence with the reference. It looks awkward and not according to scientific standards.**

**Response:** Suggestion incorporated.

**Query 5. At least mention three** **objectives and hypotheses of your current research and correlate them with the discussion section.**

**Response:** Objectives and hypotheses of research have been included in the last paragraph of the introduction.

**Query 6. Check the references in the text and the reference section. Make sure it should be according to the journal format.**

**Response:** Needful has been done.

**Query 7. I suggest you some new references related to your study. Kindly cite them in your research article which will increase the worth of your article.**

**Response:** Suggestion incorporated.

**Query 8. The** **conclusion statement should be comprehensive. Kindly revise it.**

**Response:** Conclusion statement has been revised as per suggestion.

**Query 9. The introduction looks lengthy and some of the statements are repeated, which should be avoided.**

**Response:** Needful has been done throughout the introduction as per suggestion.

**Query 10. Also, include the detailed procedure for** **Total N determination.**

**Response:** A detailed procedure of total N determination has been added under the heading of chemical analysis in detail.

**Reviewer #2:**

**Query 1. The manuscript lacks explicit presentation of control data for each experiment conducted by the authors. It is imperative to include control conditions where no organic matter was added to the soil to facilitate meaningful comparisons across different treatments. In order for the manuscript to undergo thorough review, it is essential that control data be provided for comparison. Without such data, meaningful evaluation of the experiments conducted would be compromised.**

**Response:** We appreciate your insightful comments. Since our experimental setup was soilless, it may not be appropriate to include a soil control. Therefore, we compared the available organic residues (FM, PN, and SB) in all possible combinations to fulfill the requirement for a baseline comparison. Thank you for highlighting the significance of control data. We are dedicated to offering pertinent and enlightening data to support a comprehensive assessment of the paper.
