# Supplementary material for: Utilization of organic-residues as potting media: Physico-chemical characteristics and their influence on vegetable production
Source: PLoS One. 2024 Jun 11;19(6):e0302135. doi: 10.1371/journal.pone.0302135 (PMC11166280; doi:10.1371/journal.pone.0302135)
Supplement: Attachment — Submitted filename: Response to Reviewers.docx [file pone.0302135.s002.docx]

**Response to Reviewers’ Comments**

**Reference:** PONE-D-24-02845R1

**Title:** Utilization of organic-residues as potting media: Physico-chemical characteristics and their influence on vegetable production

NOTE: Revised text as per review comments has been highlighted in the revised manuscript through track changes.

The authors sincerely appreciate the insightful comments provided by the esteemed editor and reviewers, which have greatly contributed to enhancing the quality of the manuscript. We are grateful for their valuable time and efforts dedicated to the review process. Thank you.

**Reviewer #3:**

**Query. I suggest you consider incorporating more figures than tables into your manuscript. Figures not only enhance the visual appeal of your paper but also provide a succinct and informative way to present your findings. Additionally, I encourage you to include a study area figure in your paper.**

**Response:** As per the suggestion of the respected reviewer Table 1 titled “Effect of substrate composition on spinach growth and yield attributes” (Plant height (cm), Fresh biomass (g plant^−1^), Dry biomass (g plant^−1^), Leaves plant^−1^) has been converted into figures to enhance the visual appeal. Moreover, the study area information has been provided in the materials and methods section (2.1. Site description), and the study area figure has been provided in Fig 1.
